# It is complicated: Potential short- and long-term impact of coronavirus disease 2019 (COVID-19) on antimicrobial resistance—An expert review

**DOI:** 10.1017/ash.2022.10

**Published:** 2022-02-18

**Authors:** Marco Seneghini, Susanne Rüfenacht, Baharak Babouee-Flury, Domenica Flury, Matthias Schlegel, Stefan P. Kuster, Philipp P. Kohler

**Affiliations:** 1 Division of Infectious Diseases and Hospital Epidemiology, Cantonal Hospital St Gallen, St Gallen, Switzerland; 2 Medical Research Centre, Cantonal Hospital St Gallen, St Gallen, Switzerland

## Abstract

As of December 2021, the coronavirus disease 2019 (COVID-19) pandemic has claimed millions of deaths and caused disruptions in health systems around the world. The short- and long-term effects of COVID-19 on antimicrobial resistance (AMR), which was already a global threat before the pandemic, are manifold and complex. In this expert review, we summarize how COVID-19 might be affecting AMR in the short term (by influencing the key determinants antibiotic use, infection control practices and international/local mobility) and which additional factors might play a role in the long term. Whereas reduced outpatient antibiotic use in high-income countries, increased awareness for hand hygiene, and reduced mobility have likely mitigated the emergence and spread of AMR in the short term, factors such as overuse of antibiotics in COVID-19 patients, shortage of personal protective equipment, lack of qualified healthcare staff, and patient overcrowding have presumably facilitated its propagation. Unsurprisingly, international and national AMR surveillance data for 2020 show ambiguous trends. Although disruptions in antibiotic stewardship programs, AMR surveillance and research might promote the spread of AMR, other developments could prove beneficial to the cause in the long term. These factors include the increased public awareness for infectious diseases and infection control issues, the strengthening of the One Health perspective as outlined by the Centers for Disease Control and Prevention, and the unprecedented number of international research collaborations and platforms. These factors could even serve as leverage and provide opportunities to better combat AMR in the future.

As of December 2021, the coronavirus disease 2019 (COVID-19) pandemic has affected >270 million people and has claimed >5 million lives worldwide.^1^ Consequently, healthcare systems around the globe are confronted with hitherto unmet challenges due to high COVID-19 patient numbers, lack of resources, and disruption of routine processes. Similar to COVID-19, antimicrobial resistance (AMR) represents a global public health threat that has been termed “the slow pandemic” to emphasize its more insidious increase.^2^ Infections due to resistant pathogens result in ∼700,000 deaths annually. This number has been estimated to increase to 10 million deaths annually by the year 2050.^3^

Experts have warned about the potentially deleterious effects of the COVID-19 pandemic on AMR due to the deprioritization of infection prevention and control (IPC) and antimicrobial stewardship programs (ASPs).^4^ On the other hand, factors such as social distancing and reduced international travel could potentially have favorable effects on limiting AMR prevalence.^5,6^ We compiled the available evidence on the potentially harmful but also beneficial effects of the COVID-19 pandemic on 3 key determinants of AMR: antibiotic use, IPC measures, and international mobility. We also summarize the net effect that these changes have had on AMR in the short term by compiling national and international AMR surveillance data from 2020. Finally, we discuss how the COVID-19 pandemic could affect AMR in the long term, considering potential amplifying and moderating factors.

## Antibiotic use during the COVID-19 pandemic

The main mechanisms for the development and spread of AMR are antibiotic-induced mutagenesis and selection pressure.^7^ The association between antibiotic use and the emergence of AMR has been well established, both from before and during the pandemic.^8,9^

For the inpatient setting, data on antibiotic consumption are most often expressed as defined daily doses (DDD) per number of bed days or patient days. Changes in this measure do not necessarily reflect changes in the overall antibiotic consumption if absolute patient numbers have also changed. For example, data from the Veterans’ Health Administration show an increase in the density of antimicrobial utilization (ie, antibiotic use per 1,000 patient days) in the inpatient setting for the year 2020, whereas overall antibiotic use decreased, probably due to decreases in healthcare utilization related to non–COVID-19 conditions.^10^ Similarly, national surveillance data from the United Kingdom show that total consumption (calculated as DDD per 1,000 inhabitants per day) decreased by 11% between 2019 and 2020.^11^ However, when looking at DDD per 1,000 admissions, antibiotic consumption for inpatients increased by almost 5%, reflecting changes in hospital populations since the start of the pandemic.^11^ Indeed, as many as 72% of COVID-19 patients receive antibiotic treatment, either on an empirical basis or to treat a confirmed bacterial coinfection, although bacterial coinfections are observed in only 15%–25%.^12,13^ Many of these superinfections are due to hospital-acquired infections, mostly ventilator-associated pneumonia and central-line–associated bloodstream infections.^14^ In line with these observations, antibiotic use in 17 hospitals in South Carolina increased in those institutions admitting COVID-19 patients but not in those not treating COVID-19 patients. Notably, this increase was mostly due to broad-spectrum and anti–methicillin-resistant *Staphylococcus aureus* agents.^15^ The use of antifungal substances, which has massively increased in the inpatient and particularly the ICU setting during the pandemic,^11,16^ is an important topic that is beyond the scope of this review.

For the community setting, several high-income countries (HICs) have reported reductions in antibiotic consumption in 2020. In the United States, antibiotic consumption decreased by 33%,^17^ in Canada it decreased by 26%,^18^ and in countries of the European Union it decreased by 18%.^19^ These effects have been mostly attributed to decreased consumption of antibiotics commonly used to treat children and to treat respiratory infections.^11,17–19^ This trend likely reflects the reduction in non–COVID-19 respiratory diseases that has been reported from around the globe during the pandemic, probably owing to social distancing and community lockdowns. For instance, seasonal influenza activity in 2020 was minimal in Brazil,^20^ Singapore,^21^ Japan,^22^ Taiwan,^23^ and Europe.^24^ In Australia, an impressive decrease in respiratory syncytial virus infections among children has been described.^25^ Not only viral but also bacterial pathogens with the respiratory route as primary transmission pathway have decreased substantially. Surveillance data from 26 countries have shown a sustained reduction in invasive diseases due to *Streptococcus pneumoniae*, *Haemophilus influenzae*, and *Neisseria meningitides.*^26^

Despite these favorable trends from the community setting observed in HICs, opposite trends have been reported from India, where a significant increase in nonpediatric antibiotic sales (mainly azithromycin) was observed during the first COVID-19 epidemic wave. Similar trends may have occurred in other low- and middle-income countries (LMICs),^27^ where antibiotics are commonly dispensed over the counter without a prescription.^28,29^

**Fig. 1.**
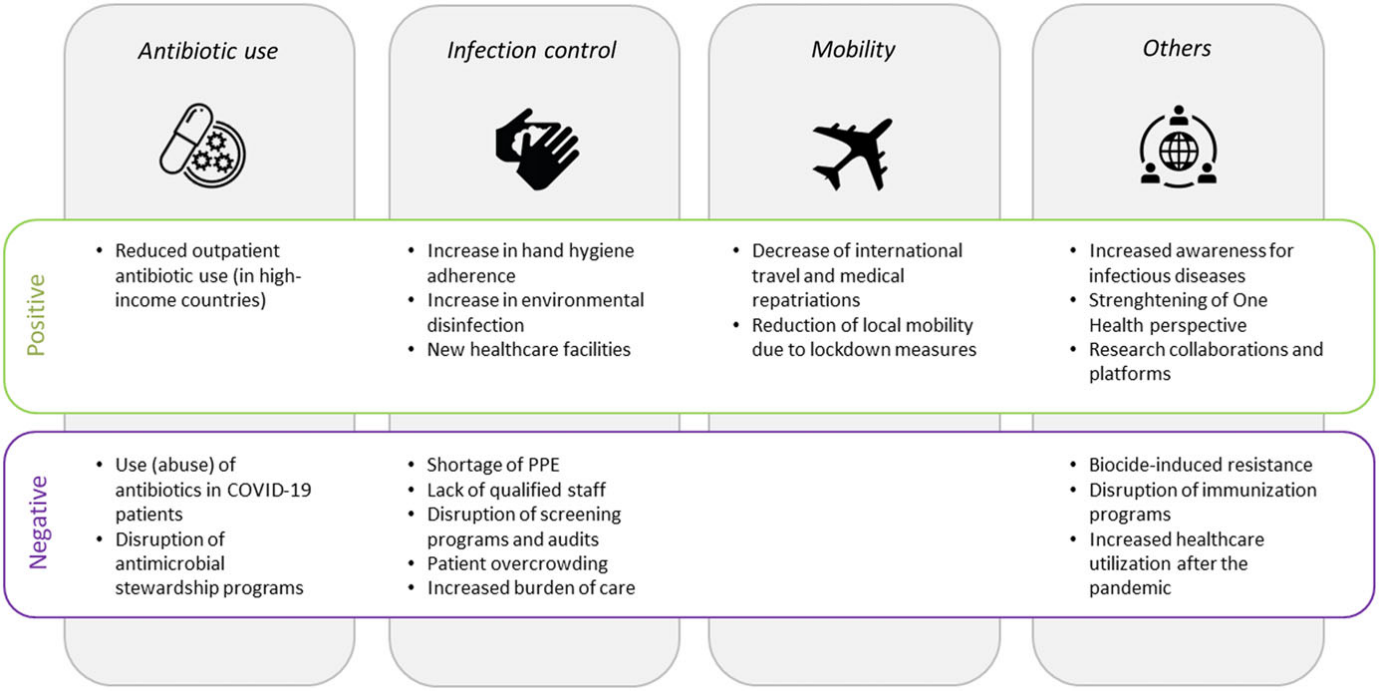
Factors associated with an increase (positive) or a decrease (negative) in antimicrobial resistance. Note. PPE, personal protective equipment.

In sum, although COVID-19 patients are often (unnecessarily) treated with antibiotic substances, overall antibiotic consumption—particularly in the community setting—has decreased during the pandemic according to several national and international surveillance reports (Fig. 1). This is mainly true for HICs, whereas data from LMICs are either lacking or show increases in use.

## Infection prevention and control

Infection prevention and control (IPC) measures are essential in controlling the spread of both COVID-19 and AMR. The contagiousness of severe acute respiratory coronavirus virus 2 (SARS-CoV-2) and the unprecedented enormous strain on hospitals have led to fundamental changes in IPC measures that have unavoidably affected the spread of multidrug-resistant organisms (MDRO) in hospitals. According to an international survey of the Global Antimicrobial Resistance and Use Surveillance System (GLASS), the quality of many IPC measures improved in several countries during the pandemic. IPC training of healthcare workers (HCWs) has been reinforced, use of personal protective equipment (PPE) was expanded, and adherence to hand hygiene (HH) has improved (Fig. 1).^30^ In a meta-analysis, HH adherence during COVID-19 was 74%, which was higher than generally estimated.^31^ For example, HH adherence increased from 46% before the pandemic to 89% during the pandemic in a study from Israel.^32^ Similarly, a study from Chicago analyzing data from automated HH monitoring revealed an increase in adherence from 55% at baseline to 76% during the pandemic.^33^ Achieved improvement, however, concomitantly decreased with decreasing SARS-CoV-2 infection rates.^33–35^ Interestingly, HH adherence has previously been described to increase during seasonal or pandemic influenza periods and could be due to increased awareness or self-protection.^36,37^

Furthermore, environmental disinfection has intensified in many places, which might also have had the benefit of eliminating unnoticed environmental MDRO sources.^38,39^ To contain an ever-increasing number of COVID-19 patients and anticipating surges, hospital admissions for non–COVID-19 patients decreased, potentially resulting in a lower number of transmissions from chronic MDRO carriers.^40^ An additional strategy adopted by many countries to increase overall patient capacity was to create “ad hoc” COVID-19 facilities or even new hospitals in record time.^41–43^ Such measures at least partly mitigated hospital overcrowding, which is a clear risk for cross transmission.^44^

On the other hand, breaches in adherence to standard IPC practices were also reported in the GLASS survey.^30^ Indeed, MDRO outbreaks have frequently been reported from COVID-19 wards. Particularly common were upsurges of *Candida auris* and carbapenem-resistant organisms (CROs), which could be at least partly ascribed to decreased adherence to IPC practices.^45–58^ A formative research study from 2020 underlined stress and busyness as potential barriers to correct HH performance.^59^ Similarly, longer duty hours and suboptimal HH were associated with SARS-CoV-2 acquisition among HCWs.^60^ PPE shields HCWs from infection, and gloving hands paradoxically can reduce HH compliance,^61,62^ which has been revealed during the ongoing pandemic.^35^ For instance, in a carbapenem-resistant Enterobacterales (CRE) outbreak report from an Italian ICU, COVID-19 patients needing ventilation in the prone position were more likely to acquire CRE. Furthermore, contamination of gloves and gowns of HCW involved in patient positioning might have contributed to CRE transmission.^63^

Another factor often mentioned in the context of MDRO outbreaks is PPE shortages, which have affected both HICs and LMICs.^64–66^ In addition, lack of supllies might have led in some cases to the practice of sharing or reusing PPE, which also increases the risk of cross transmission.^67^ An additional factor contributing to disruption in IPC measures is the shortage of healthcare staff. Reasons for staff shortages are (1) HCWs on sick leave, as frontline HCWs have at least a 3-fold increased risk of acquiring SARS-CoV-2 compared to the general community^68^; (2) sick leave due to conditions other than COVID-19, for instance, due to burnout or other mental illnesses which have frequently been reported among HCW during the pandemic^69^; (3) HCWs in quarantine as a consequence of SARS-CoV-2 positive household contacts; and (4) a relative shortage due to increased need of care for COVID-19 patients.^70^ Indeed, a critically low HCW-to-patient ratio is a well-established risk factor for the transmission of MDROs, as shown for MRSA.^71^ Also, newly recruited staff without work experience in ICU settings and without the necessary competences in IPC practices have been mentioned as factor contributing to MDRO outbreaks.^63^ Other collateral damage of the pandemic that has negatively affected AMR includes the suspension of routine PPE- and hand hygiene audits and the interruption of MDRO admission screening programs (Fig. 1).^58,72^

## Mobility and travel restrictions

The spread of MDROs is highly facilitated by international and local travel. Travelers to LMICs with high AMR prevalence are at particular risk to become colonized with resistant, mostly gram-negative bacteria.^73,74^ For instance, up to 80% of travelers to South Asia are at least transiently colonized with ESBL-producing Enterobacterales.^75,76^ Once colonized, >10% remain colonized for 12 months, and 12% will transmit the pathogen to another household member.^76^ The role of leisure tourism in the spread of MDROs is still likely to be underestimated because returning travelers are not routinely tested for resistant bacteria unless they exhibit symptoms.^77^ Travelers with healthcare contact abroad are at a high risk to be colonized or infected with even more resistant pathogens, including carbapenem-producing organisms.^78,79^ Also, patients undergoing aeromedical evacuation have a higher risk of being colonized with methicillin-resistant *S. aureus* (MRSA) or multidrug-resistant gram-negative bacilli.^80^ Aeromedical evacuations of such patients from countries with a high burden of AMR have resulted in many nosocomial outbreaks in low-endemicity countries.^81–83^

Many countries started to impose travel bans and quarantine restrictions due to COVID-19, and a large decrease in international air travel was predicted for 2020.^84^ Similarly, as a consequence of the travel restrictions, a decrease of 44% in medical repatriations in the year 2020 were predicted in Switzerland according to the annual report of the Swiss Air-Rescue.^85^ As a result of such a reduction in normal travel patterns and medical repatriations, a decrease in the prevalence of carriage with mainly ESBL-producing Enterobacterales and lower importation of high-risk pathogens into low-endemicity countries is likely.^86^

Lockdown measures during COVID-19 affected not only international travel but also local mobility. According to Google mobility reports, a reduction in human movement, along with mitigation of SARS-CoV-2 spread, occurred in 2020.^87,88^ Although the role of local mobility for the acquisition of resistant pathogens is unclear, the reduction of human contacts accompanying reduced mobility might decrease the spread of some pathogens. At least for MRSA, detection in public transportation and other public spaces has been reported, which could definitely point toward a lower risk of MDRO acquisition with decreased local mobility.^89–93^

In sum, decreased international travel and local mobility can be expected to reduce the burden of AMR (Fig. 1). This trend might be particularly true for low-endemicity countries, where importation of resistant pathogens is a relevant contributor to the local burden of AMR. However, the positive impact of such measures will most likely subside as soon as mobility restrictions are lifted and international travel has returned to prepandemic levels.

## Net effect on AMR in 2020

Surveillance data from Europe for 2020 suggest that the COVID-19 pandemic has affected AMR in many ways.^94^ In countries of the European Union, most bacterial species–antimicrobial group combinations show a decreasing or stabilizing trend in 2020 including third-generation cephalosporin-resistant *Escherichia coli.*^94^ Similarly, data from the United Kingdom show that the burden of antibiotic resistance declined in 2020, largely due to a decrease in *E. coli* bloodstream infections.^11^ These trends could indeed be explained by the massive reduction of international travel, which is associated with acquisition of ESBL Enterobacterales.

Whereas community-acquired pathogens, such as *E. coli* or *S. pneumoniae*, have been less frequently reported in 2020, typical healthcare pathogens, such as *Acinetobacter* spp or *Enterococcus faecium*, were more frequently observed, according to data from the Central Asian and European Surveillance of Antimicrobial Resistance (CAESAR) network. Similar results have been reported from the European Antimicrobial Resistance Surveillance Network (EARS-Net).^94^ Also, alerts have been issued on the emergence of CPE from Latin America and the Caribbean region.^95^ Contrasting with these trends, the number of CPE isolates decreased in Switzerland in 2020.^96^ Factors that could explain these discrepancies between countries include the local endemicity of specific resistant pathogens and the different strain on healthcare systems caused by COVID-19.

As with antibiotic consumption data, AMR surveillance data must be interpreted with careful consideration of the denominator.^97^ Also, screening for high-risk pathogens, such as CPE or VRE, stopped in certain low-risk settings.^11^ Furthermore, resources for AMR surveillance programs have been shortened or reassigned to other areas during the pandemic, as reported by most of the 73 countries participating in the GLASS survey. Particularly in LMICs, reduced availability of laboratory reagents to detect resistant pathogens has been reported.^30^

## Long-term impact of COVID-19 on AMR

How the COVID-19 pandemic might affect AMR in the long term remains unclear.^98^ The pandemic might facilitate further propagation of AMR, although many unintended consequences might also help to mitigate AMR (Fig. 1). Early in the pandemic, experts warned that disruption of antimicrobial stewardship during the pandemic could result in further expansion of AMR.^99^ Indeed, in a before-and-after study from Naples, Italy, antibiotic consumption increased after the interruption of an ASP, mainly in surgical wards and in wards where the ASP was less established.^100^ To prevent such effects, integration of antimicrobial stewardship specialists into disaster preparedness plans has been advocated,^72^ and suggestions have been made regarding how antimicrobial stewardship activities could be refocused in the light of the COVID-19 pandemic.^101^ In fact, several hospitals have reported successful adaptation of their ASP to the COVID-19 response.^102,103^ How these interruptions and modifications of ASP affect antibiotic consumption and how this impacts on AMR in the long term remains to be seen.

Elective surgeries and chemotherapies for cancer patients had to be delayed during the pandemic.^104–106^ These delays have potentially contributed to a reduced healthcare utilization and also to reductions in antibiotic prescribing. Due to the accumulated demand for these interventions in the near future, a rebound effect is likely in the sense that these interventions will add pressure to healthcare systems and will eventually increase antimicrobial use anew.^107^ Again, the availability of institutional ASPs will be critical to controlling this scenario.

Another aspect of the pandemic is the massive increase in the use of biocides, defined as compounds with antiseptic, disinfectant, or even preservative capacities^108^ in both community and hospital settings.^109,110^ Questions regarding their possible impact on AMR, including cross resistance to unrelated antimicrobials, have been raised.^111^ Biocides can lead to AMR in many ways: membrane modification, upregulation of efflux pumps,^112^ the increase of the propensity to form biofilms,^113^ or even the induction of a viable but nonculturable state that permits survival in unfavorable environmental conditions.^114^

However, due to insufficient investigative data and lack of standardized protocols, the full magnitude of the possible impact of biocides on AMR in the long term remains difficult to assess.^113^ In general, the One Health aspect outlined by the Centers for Disease Control and Prevention has gained considerable attention during the pandemic. The origin of SARS-CoV-2 is suspected to have occurred at the interface among humans, animals, and the environment.^115^ Several factors facilitating the spread of SARS-CoV-2 have been discussed such as climate change, growing populations, mobility, and global trade. International initiatives have been launched to address these issues and to be prepare for emerging zoonotic diseases.^116^ Strengthening the One Health perspective will most likely have a positive impact on limiting AMR in the long term.^117^

From a global health perspective, the disruption of immunization programs—especially in LMICs—harbors the risk for major long-term consequences. Data from Pakistan and Saudi Arabia show that routine immunization of children was considerably impaired in 2020, making this vulnerable population susceptible to diseases that may require antibiotic treatment.^118,119^ Notably, US national authorities also reported a decline in routine pediatric vaccine ordering early in the pandemic.^120,121^

The COVID-19 pandemic has revolutionized our idea of international collaborations and data exchange, particularly regarding genomic surveillance.^122^ A multitude of international research groups have been established to study the epidemiology, risk factors and therapeutic aspects of COVID-19.^123^ These newly created infrastructures and platforms provide excellent research opportunities to study and tackle other infectious diseases including drug-resistant pathogens.^124,125^

On the societal level, awareness and sensitization regarding infectious diseases and IPC has been boosted by the COVID-19 pandemic.^124^ This awareness could reinforce the role of these disciplines within hospitals and healthcare systems, increase chances for research funding, and facilitate support from public health authorities.

An important limitation of this review is the lack of data from LMICs, where the consequences of COVID-19 on AMR might be even more pronounced due to preexisting inequalities, including weak or nonexisting antibiotic regulations, insufficient infrastructure, technological gaps, and inadequate health coverage.^126^ Therefore, a better understanding of the inter-relatedness of COVID-19 and AMR might be of particular importance for LMICs.

In conclusion, the ways in which the COVID-19 pandemic has affected and will influence AMR are manifold. For the hospital setting, high antibiotic consumption in COVID-19 patients and several MDRO outbreaks during the pandemic have been reported; however, national AMR surveillance data show ambiguous trends. For the community setting, surveillance data show decreases in antibiotic consumption and in AMR for the year 2020, at least for HICs. Differences between countries with prepandemic low and high AMR burdens and limitations arising from the disruption of AMR surveillance programs (particularly in LMICs) have to be considered when interpreting these findings. The long-term impact of the pandemic on AMR remains unclear. However, the increased public awareness for infectious diseases and for One Health issues in the wake of the pandemic, and the unprecedented intensity of international research networks and collaborations can provide leverage and opportunities to better combat AMR in the near future.
